# Comprehensive records of collected tick species data in Japan

**DOI:** 10.1016/j.dib.2025.112000

**Published:** 2025-08-21

**Authors:** Kaori Morishima, Hayato Iijima, Kandai Doi, Hirotaka Komine, Yuya Watari, Shun’ichi Makino, Kimiko Okabe

**Affiliations:** a Sakushin Gakuin University Woman’s College. 908, Takeshitamachi, Utsunomiya, Tochigi 321-3295, Japan; b Forestry and Forest Products Research Institution. 1, Matsunosato, Tsukuba, Ibaraki 305-8687, Japan; c Yamagata University. 1-23, Wakaba-machi, Tsuruoka, Yamagata 997-0037, Japan

**Keywords:** Geographical distribution, Host animal species, Ixodidae, Parasite

## Abstract

We collected original papers, abstracts of academic conference speeches, research reports of university hospitals and private clinics, bulletins of science museums, and reports from prefectural health institutions containing tick sampling information published from 1911 to 2021 using bibliography in journals published by the Japanese Association for Acarology and the Acarological Society of Japan. We also searched such publications through Google scholar using scientific and Japanese names of 46 tick species. We listed the recorded tick names, collected years, prefectures, the islands, survey months, collected month, tick developmental stages (larva, nymph, and adult), collection method (flagging from vegetation or direct sampling from host animal), and host animal species. This dataset may be useful to analyse trend and changes in the distribution of ticks correlated with environmental factors such as land use and climate.

Specifications Table

**Table:** 

Subject	Biology
Specific subject area	Ecology, Epideminology, Entomology
Type of data	Table, Raw
Data collection	We collected original papers, abstracts of academic conference, research reports of university hospitals and private clinics, bulletins of science museums, and reports from prefectural health institutions containing tick sampling information published from 1911 to 2021 using bibliography published by the Japanese Association for Acarology and the Acarological Society of Japan. We also searched such publications through Google scholar using scientific names and Japanese names of 46 tick species. We listed the recorded tick names, collected years, the prefecture, the island, survey months, occurrences month, tick developmental stage (larva, nymph, and adult), collection method (flagging from vegetation or direct sampling from host animal), and host animal species. We confirmed the tick species names of synonyms based on reliable literatures. We revised the misidentification of the four tick species.
Data source location	The study sites are spread over the geographic area from 24.00 to 46.00 °N in latitude and from 123.00 to 146.00 °E in longitude (geographic coordinate system is WGS84).
Data accessibility	Repository name: Zenodo, Biodiversity Literature Data identification number: https://doi.org/10.5281/zenodo.15278563 Direct URL to data: https://zenodo.org/records/15,278,563
Related research article	none

## 1 Value of the Data

• The data provide the location and timing of collection of tick species in Japan from 1911 to 2021. The data included many records that were written only in Japanese. Such non-English records have never been used and available for non-Japanese researchers.
• The data benefit ecologists, environmental scientists, statisticians, environmental health researchers, public health researchers, epidemiologists, and medical workers who could utilize the data to gain further insights on how local-scale natural environments influence the questing activities of ticks. The data are valuable for wildlife and veterinary scientists investigating host-parasite interactions, particularly in relation to wildlife, which serve as important hosts for several tick species.
• This data could be useful for analysing changes in tick distribution in relation to environmental factors, such as land use and climate, as well as for assessing the risk of tick-borne diseases.

## 2 Background

The objective of this dataset is to provide the comprehensive information about distribution, seasonality and hosts of collected tick species in Japan. Emerging infectious diseases have been a global burden due to the rapid expansion of infection consequent upon land-use changes and modern transport since the late 20th century [1,2]. Among them, 20 to 30 % of them are suspected vector-borne diseases, including mosquito-borne and tick-borne [3,4]. The global distribution of both vector arthropods is expanding as host animals and commodities migrate [5]. Infected areas of vector-borne diseases are also related to recent biodiversity loss and climate change [6,7].

In response to this growing concern, existing data collection related to infectious disease vectors [8] and the development of access to such data is crucial to analyze current and future vector-borne disease risks. Japan has information on infected areas and the number of occurrences of tick-borne diseases including the Japanese spotted fever (JSF), Lyme disease, tick-borne encephalitis, severe fever with thrombocytopenia syndrome (SFTS) [9], and Turalemia (https://id-info.jihs.go.jp/, Accessed 7 May 2025). Furthermore, including information that were written by non-English language enhances data completeness and reduces knowledge gaps in recent years [10].

## 3 Data Description

The data consists of three spread sheets [11]. They are TickLiteratureDB.csv, TickCollectDB.csv, and TickSpeciesList.csv. The first one is the literature information that we collected (Table 1), the second one is the extracted information from these literatures (Table 2), and the third one is all tick species list that are present in TickCollectDB.csv (Table 3).

**Table 1:** Variables in TickLiteratureDB.csv.

Variable	Explanation	Note
ResourceID	ID of each literature	
Author	Author(s)’ name	Family name Given name.
Year	Year of publication	
Title	Title of the literature	
Journal	The name of journal	
Volume	The volume number of the literature	
Pages	The first and end page number of the literature	First page-end page.

**Table 2:** Variables in TickCollectDB.csv.

Variable	Explanation	Note
Modified	The date when we confirmed the information	YYYY/MM/DD
ResourceID	ID of each literature	The IDs correspond to IDs in TickLiteratureDB.csv
JapaneseName	Japanese name of collected tick species	
ScientificName	Scientific name of collected tick species	
Stage	The developmental stage of collected tick	Coded string: “A” is adult, “N” is nymph, and “L” is larva.
Year	Year of collection	If the sampling period encompasses several years and the author did not specify any year, the first and end years were written.
MonthSurvey	The month when survey was conducted	
Month	The month when tick was collected	
Prefecture	The name of prefecture of sampling location	
Island	The name of island in Japan of sampling location	
Method	Tick sampling method	Coded string: “medical examination” is the records from human bites. “veterinary examination” is the records from livestock by veterinarian. “field survey” is the records including flagging and wildlife and livestock’s sampling. “accidental collection” is the record that ticks are collected accidentally.
Host/Habitat	The scientific names of tick hosts and the type of habitat where ticks are collected	“vegetation” is the records by flagging. “litter” and “soil” are the records by soil sampling. “mammals” and “birds” are the records from unidentified animalsß.

**Table 3:** Variables in TickSpeciesList.csv.

Variable	Explanation	Note
Scientific name		
Japanese name		
notes	The information about past identification, synonyms, and any other remarks about species names.	
referemce ID	The basis of notes.	The IDs correspond to IDs in TickLiteratureDB.csv

## 4 Experimental Design, Materials and Methods

We collected original papers, abstracts of academic conference, research reports of university hospitals and private clinics, bulletins of science museums, and reports from prefectural health institutions containing tick sampling information published from 1911 to 2021 using bibliography published by the Japanese Association for Acarology and the Acarological Society of Japan. We also searched such publications through Google scholar using scientific names and Japanese names of 46 tick species [12]. However, for *Haemaphysalis flava* and *H. longicornis*, we stopped search through Google scholar after checking the top 100 searched articles because there are huge number of articles on these species and most of the articles after the first 100 were duplicates with bibliographies and/or information without peer reviews.

Recorded tick names, collected years, the prefecture, and the island were compiled per row into TickCollectDB.csv. For consecutive sampling across years, we used data on which the sampling year of a specific species was described. Additionally, we also compiled survey months, occurrences month, tick developmental stage (larva, nymph, and adult), collection method (flagging from vegetation or direct sampling from host animal), and host animal species into TickCollectDB.csv. If these information is not able to be obtained from the literature, we record it as “NA”.

We confirmed and corrected the tick species names (both of scientific name and Japanese name) based on literatures. These names and the basis of them are described in the TickSpeciesList.csv. The scientific name of host mammals was conformed on Ohdachi et al. [13]. and the list of Japanese common name of the world’s mammals (https://www.mammalogy.jp/list/index.html, accessed on 29 Jul 2025). The scientific name of host birds was conformed on a checklist of Japanese birds [14] and IOC World Bird List (v15.1, https://www.worldbirdnames.org/new/, accessed on 29 Jul 2025). The scientific names of host reptiles and amphibians were conformed on a list by Herpetological Society of Japan (https://herpetology.jp/wamei/, accessed on 29 Jul 2025).

We included the records that had either the collected year or the collected location in TickCollectDB.csv. We excluded *Haemaphysalis megalimae* because the host record was questionable.

When a publication listed either a Japanese or scientific name, we added the other to our database based on TickSpeciesList.csv. When a Japanese or scientific species name used in a publication was not listed in TickSpeciesList.csv, we excluded it from our TickCollectDB.csv. We did not include morphological species in TickCollectDB.csv.

## Limitations

As already stated, our dataset may not cover all of the records of *H. flava* and *H. longicornis* because we did not check all hits by google scholar about these species. Furthermore, our data set does not include records published after 2021.

## Ethics Statement

The authors have read and follow the ethical requirements for publication in Data in Brief and confirming that the current work does not involve human subjects, animal experiments, or any data collected from social media platforms.

## CRediT Author Statement

**Kaori Morishima:** Investigation, Writing - Original Draft; **Hayato Iijima:** Data Curation, Writing - Review & Editing; **Kandai Doi:** Investigation, Writing - Review & Editing; **Hirotaka Komine:** Investigation, Writing - Review & Editing; **Yuya Watari:** Investigation, Writing - Review & Editing; **Shun’ichi Makino:** Investigation, Writing - Review & Editing; **Kimiko Okabe:** Conceptualization, Project administration, Investigation, Writing - Review & Editing.

## Data Availability

ZenodoJuly 30, 2025 (2) Dataset Open Records of collected tick in Japan from 1911 to 2021 (Original data).
